# Gall bladder schistosomiasis diagnosed incidentally post laparoscopic cholecystectomy: A case report

**DOI:** 10.1016/j.ijscr.2023.108787

**Published:** 2023-09-02

**Authors:** Willbroad Kyejo, Sajida Panjwani, Allyzain Ismail, Blessing Mathew, Caroline Ngimba, Ally Mwanga

**Affiliations:** a The Aga Khan University, East Africa Medical college, Tanzania; b Department of General Surgery, The Aga Khan Hospital, Dar-es-Salaam, Tanzania; c Department of Pathology, The Aga Khan Hospital, Dar-es-Salaam, Tanzania; d Department of Surgical Gastroenterology, Muhimbili University of Health and Allied Sciences, Dar-Es-Salaam, Tanzania

**Keywords:** Gallbladder schistosomiasis, Neglected tropical disease, Rare entity, Case report

## Abstract

**Introduction and importance:**

Schistosomiasis is a neglected tropical disease caused by parasitic worms of the genus Schistosoma. It primarily affects the intestines, liver, and urinary tract however, rare cases have been reported where the parasite invades other organs. This case report presents an incidental finding of schistosomiasis, upon histopathology evaluation, in a patient who underwent laparoscopic cholecystectomy for symptomatic gallstone disease with recurrent attacks of cholecystitis.

**Case presentation:**

We present the case of a 42-year-old female patient who presented to the emergency department with symptoms and signs suggestive of acute cholecystitis. She underwent conservative management with interval laparoscopic cholecystectomy with histopathology findings of Schistosoma eggs within the walls of the gallbladder. Underwent eradication therapy with praziquantel.

**Clinical discussion:**

The finding of Schistosoma eggs in the gallbladder wall during routine histopathological examination highlights the importance of considering schistosomiasis, and other parasites, in cases of recurrent bouts of cholecystitis. The case challenges the conventional understanding of the transmission patterns of this parasitic infection and raises questions about potential atypical life cycle routes within the human body. It also emphasizes the importance of routine histopathology analysis of specimen removed from the body.

**Conclusion:**

This case report presents a rare occurrence of schistosomiasis cholecystitis in a 42-year-old female patient underscoring the importance of considering parasitic infections. Thorough histopathological examination in routine surgeries is crucial for early detection and targeted treatment. The patient's positive response to praziquantel therapy highlights its effectiveness in managing schistosomiasis, which is a neglected tropical disease.

## Highlights

• Rarity of gall bladder schistosomiasis
• Importance of suspicion for parasitic causes of recurrent cholecystitis
• Significance of routine histopathological analysis of specimen
• Role of praziquantel in extraintestinal schistosomiasis manifestations

## 1 Introduction and importance

Schistosomiasis, also known as bilharzia, is a neglected tropical disease (NTD) caused by parasitic worms of the genus Schistosoma [1]. It is prevalent in various tropical and subtropical regions, particularly in areas with inadequate sanitation and contaminated freshwater sources [1,2]. The disease affects millions of people worldwide, with a significant burden on public health and socioeconomic development [1,3].

While schistosomiasis primarily affects the intestines, liver, and urinary tract, rare cases have been reported where the parasite invades other organs, including the gallbladder [4]. Schistosomiasis cholecystitis, the involvement of the gallbladder, is an unusual and infrequently encountered manifestation of the disease [4,5]. The diagnosis of gallbladder schistosomiasis prior to laparoscopic cholecystectomy, especially in non-endemic areas, adds an extra layer of complexity to this already intriguing scenario [1,6].

This case report presents an incidental finding of schistosomiasis, upon histopathology evaluation, in a patient who underwent laparoscopic cholecystectomy for symptomatic gallstone disease with recurrent attacks of cholecystitis. The finding of Schistosoma eggs in the gallbladder wall during routine histopathological examination highlights the importance of considering schistosomiasis, and other parasites, in cases of recurrent bouts of cholecystitis. The case challenges the conventional understanding of the transmission patterns of this parasitic infection and raises questions about potential atypical life cycle routes within the human body.

In this paper, we aim to shed light on the clinical presentation, diagnostic workup, management, and follow-up of the patient diagnosed with schistosomiasis cholecystitis. We also discuss the potential mechanisms of infection and emphasize the significance of vigilant examination and comprehensive evaluation during routine surgical procedures. Furthermore, this case underscores the importance of maintaining a high index of suspicion for parasitic infections, even in non-endemic areas, and the need for continued efforts in global surveillance, research, and awareness to combat this overlooked public health challenge.

By reporting and analyzing such atypical cases, we hope to contribute to the existing literature on schistosomiasis and raise awareness among healthcare professionals about the possible manifestations of the disease beyond its classical presentation. Understanding the complexities of schistosomiasis transmission can aid in early diagnosis, timely treatment, and prevention measures, ultimately reducing the burden of this NTD and improving patient outcomes. This paper has been reported in line with the SCARE 2020 criteria [7]. This article has been registered with the Research Registry.

## 2 Case presentation

A 42-year-old female patient presented with recurrent episodes of right upper quadrant (RUQ) abdominal pain and dyspepsia for the past six months. Had previously been treated at multiple periphery hospitals with analgesia and antibiotics attaining temporary relief before another bout of RUQ pain. At presentation had persistent RUQ pain for 5 days, worsened with fat rich meals and associated with nausea and vomiting. On examination had localized tenderness in the RUQ and epigastric region with a positive Murphy's sign. Laboratory tests revealed elevated inflammatory markers and abdominal ultrasound showing features of calculous cholecystitis. Due to duration of symptoms was initiated on conservative approach and upon resolution was scheduled for an interval cholecystectomy. The laparoscopic cholecystectomy was uneventful and the gallbladder was removed intact. The patient had an uncomplicated postoperative recovery and was discharged on the second postoperative day.

**Fig. 1:**
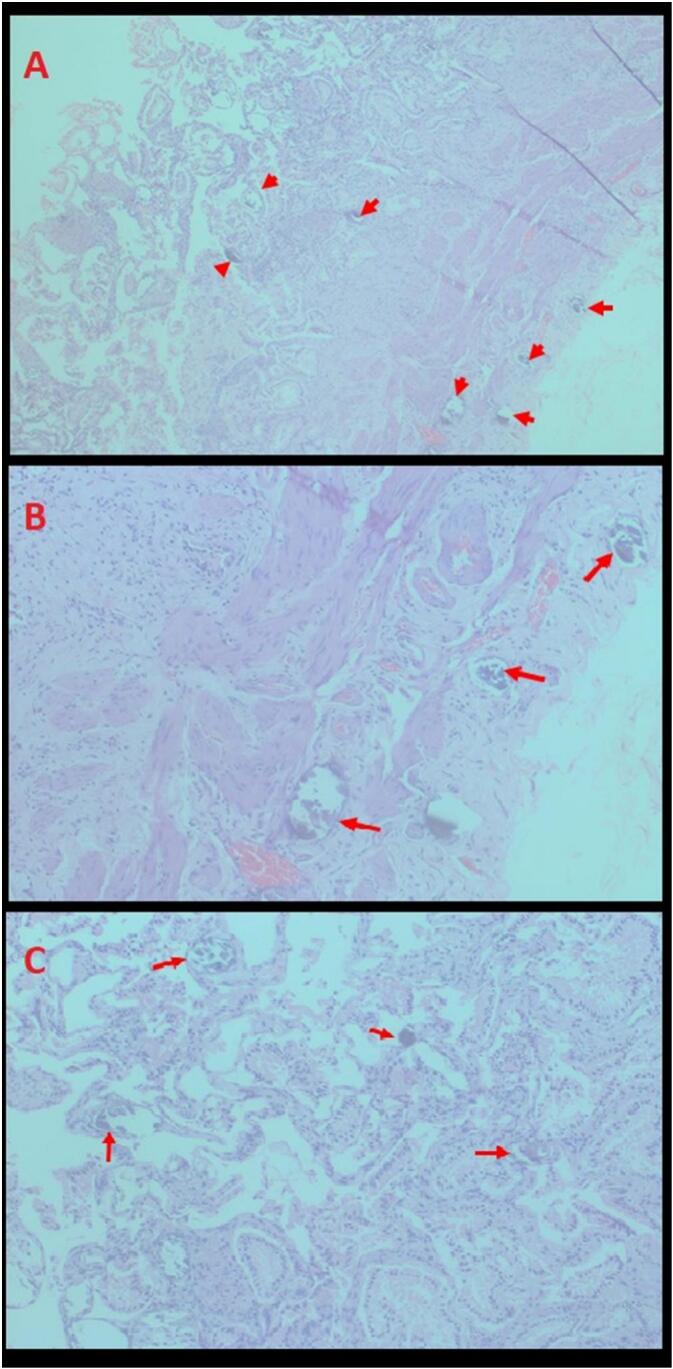
Hematoxylin and eosin stain under microscopy showing calcified Shistosoma eggs (red arrows). A – ×10 magnification shows calcified Schiostoma eggs at lamina propria and muscularis propria. B – ×20 magnification shows calcified schiostoma eggs in muscularis propria. C – ×20 magnification shows calcified schiostoma eggs in the lamina propria of the gallbladder. (For interpretation of the references to colour in this figure legend, the reader is referred to the web version of this article.)

Routine histopathological examination of the excised gallbladder revealed the presence of scattered calcified Schistosoma eggs noted within the lamina propria and muscularis propria of the gallbladder (Fig. 1). The eggs were suspected to be of *Schistosoma mansoni* based on their morphology, size, and shape. Other findings included smooth serosa, presence of few black stones, benign mucosal glands and Rokitansky Aschoff sinuses.

Given the unexpected finding of Schistosoma eggs in the gallbladder, further investigations were initiated to determine the origin of the infection. The patient had no recent history of international travel or exposure to freshwater sources associated with schistosomiasis. However, reported during her teenage years of swimming infrequently in the Lake Victoria which is one of the recognized hotspots of schistosomiasis worldwide. She could not recall experiencing overt symptoms of schistosomiasis.

Serological tests for schistosomiasis were conducted, and the results confirmed the diagnosis. It was suspected that the patient might have acquired the infection during her teenage years by swimming in the Lake Victoria, or possibly via contaminated water bodies in the local region later on in life.

Following the diagnosis of schistosomiasis, the patient was referred to an infectious disease specialist for further evaluation and management. Treatment with praziquantel was initiated, and the patient showed response during therapy. Repeat serological tests confirmed a decline in schistosomiasis-related antibodies as well as negative urine and stool microbiology and is scheduled for 3 monthly follow up to ensure eradication.

## 3 Discussion

Schistosomiasis is a NTD caused by parasitic worms of the genus Schistosoma [1]. It affects millions of people worldwide, primarily in the tropical and subtropical regions with inadequate sanitation and contaminated freshwater sources [1,2]. Schistosomiasis commonly affects the intestines, liver, and urinary tract, but its involvement in other organs, such as the gallbladder, is rare [4]. In this case, we present a unique incidentally diagnosed schistosomiasis cholecystitis in a 42-year-old female patient.

The patient's clinical presentation of recurrent RUQ abdominal pain and dyspepsia raised suspicion of cholecystitis confirmed on ultrasound and underwent interval elective laparoscopic cholecystectomy. However, the unexpected finding of Schistosoma eggs during routine histopathological examination of the excised gallbladder raised several questions regarding the origin and mode of transmission of the parasite. The patient had no recent history of travel to endemic regions with only exposure to known sources of schistosomiasis more than 20 years ago during her teenage years. She resided in a non-endemic area and had no high-risk behavior that could explain the infection. This observation suggests the possibility of schistosomiasis transmission during her teenage years or in a non-endemic region through undocumented exposure or an atypical route.

To confirm the diagnosis, serological tests for schistosomiasis were conducted and the results were positive, supporting the presence of an active schistosomiasis infection. The patient was promptly referred to an infectious disease specialist for further evaluation and management. Treatment with praziquantel, the drug of choice for schistosomiasis, was initiated, leading to a favorable therapeutic response. The successful treatment outcome in this case indicates the importance of early diagnosis and timely initiation of appropriate therapy. Praziquantel is known to effectively target the adult worms and is considered safe and well-tolerated in most patients [8]. As evidenced by the patient's clinical improvement and decline in schistosomiasis-related antibodies, the treatment successfully reduced the parasite burden.

The incidental diagnosis of schistosomiasis cholecystitis in a non-endemic area raises several intriguing possibilities [9]. It is essential to consider alternative routes of transmission and potential atypical sources of infection [10]. While the exact mechanism of transmission remains unclear, accidental exposure to contaminated water bodies or unrecognized migration patterns could play a role [4].

Moreover, this case emphasizes the importance of thorough histopathological examination of excised tissues, even in routine surgical procedures. Incidental findings of parasitic infections, such as schistosomiasis, may lead to early diagnosis and targeted treatment, thereby preventing potential complications and improving patient outcomes.

Despite the rarity of schistosomiasis cholecystitis in non-endemic areas, this case underscores the need for healthcare professionals to remain vigilant and consider parasitic infections as differential diagnoses, especially when patients present with recurrent symptoms or unusual findings [11]. Global surveillance and awareness efforts are essential to track the prevalence and distribution of parasitic diseases, including schistosomiasis, and to develop appropriate preventive measures in both endemic and non-endemic regions [[1], [2], [3]].

## 4 Conclusion

This case report presents a rare occurrence of schistosomiasis cholecystitis in a 42-year-old female patient without a history of travel to endemic regions. The diagnosis was incidental following a laparoscopic cholecystectomy, underscoring the importance of considering parasitic infections even in non-endemic areas. Thorough histopathological examination in routine surgeries is crucial for early detection and targeted treatment. The patient's positive response to praziquantel therapy highlights its effectiveness in managing schistosomiasis. Further research is needed to understand transmission in non-endemic regions. Vigilance among healthcare professionals is essential to improve patient outcomes and combat NTD.

## Patient's perspective

I could not believe worm eggs were found within my body and was disgusted at first. However, I was in discomfort for more than 6 months and since my operation I feel much better. I have not felt the pain since hence am very grateful for the care involved.

## Informed consent

Verbal informed consent was obtained from the patient for the anonymized information to be published in this article. Written consent is not required at our institution (Aga Khan University, Tanzania) for case reports if patient particulars are not disclosed in the write up or during use of images.

## Ethical approval

Ethical approval for this study (Ethical Committee Ref. AKU/08/15/338F) was provided by the Ethical Committee NAC of Aga khan University Dar es salaam, Tanzania on 16 July 2023.

## Funding

This research did not receive any specific grant from funding agencies in the public, commercial, or not-for-profit sectors.

## Author contribution

W.K: Study conception, production of initial manuscript, collection of data, proofreading.

S.P: Revision of the manuscript, proofreading.

A.I: Revision of the manuscript, proofreading.

B.M: Revision of the manuscript, proofreading.

C.N: Production of initial manuscript, collection of data.

A.M: Study conception, production of initial manuscript, collection of data.

## Guarantor

Dr. Ally Mwanga.

## Research registration number

researchregistry9406

## Provenance and peer review

Not commissioned, externally peer-reviewed.

## Conflict of interest statement

No conflicts of interest.
